# Associations of Arginine with Gestational Diabetes Mellitus in a Follow-Up Study

**DOI:** 10.3390/ijms21217811

**Published:** 2020-10-22

**Authors:** Izabela Burzynska-Pedziwiatr, Adrian Jankowski, Konrad Kowalski, Przemyslaw Sendys, Andrzej Zieleniak, Katarzyna Cypryk, Monika Zurawska-Klis, Lucyna A. Wozniak, Malgorzata Bukowiecka-Matusiak

**Affiliations:** 1 Chair of Medical Biology, Laboratory of Metabolomic Studies, Department of Structural Biology, Faculty of Medicine Department of Biomedical Sciences, Medical University of Lodz, Zeligowskiego 7/9, 90-752 Lodz, Poland; izabela.burzynska-pedziwiatr@umed.lodz.pl (I.B.-P.); adrian.jankowski@stud.umed.lodz.pl (A.J.); andrzej.zieleniak@umed.lodz.pl (A.Z.); 2 Masdiag Diagnostic Laboratory, Żeromskiego 33, 01-882 Warszawa, Poland; konrad.kowalski@masdiag.pl (K.K.); przemyslaw.sendys@masdiag.pl (P.S.); 3 Department of Diabetology and Internal Diseases, Medical University of Lodz, Pomorska 251, 92-213 Lodz, Poland; katarzyna.cypryk@umed.lodz.pl (K.C.); monika.zurawska-klis@umed.lodz.pl (M.Z.-K.)

**Keywords:** GDM, arginine, follow-up studies, T2DM risk

## Abstract

In the reported study we applied the targeted metabolomic profiling employing high pressure liquid chromatography coupled with triple quadrupole tandem mass spectrometry (HPLC–MS/MS) to understand the pathophysiology of gestational diabetes mellitus (GDM), early identification of women who are at risk of developing GDM, and the differences in recovery postpartum between these women and normoglycemic women. We profiled the peripheral blood from patients during the second trimester of pregnancy and three months, and one year postpartum. In the GDM group Arg, Gln, His, Met, Phe and Ser were downregulated with statistical significance in comparison to normoglycemic (NGT) women. From the analysis of the association of all amino acid profiles of GDM and NGT women, several statistical models predicting diabetic status were formulated and compared with the literature, with the arginine-based model as the most promising of the screened ones (area under the curve (AUC) = 0.749). Our research results have shed light on the critical role of arginine in the development of GDM and may help in precisely distinguishing between GDM and NGT and earlier detection of GDM but also in predicting women with the increased type 2 diabetes mellitus (T2DM) risk.

## 1. Introduction

Gestational diabetes mellitus (GDM) occurs for the first-time during pregnancy, is diagnosed between the second and the third trimester of gestation and characterized by glucose intolerance. The instances of gestational diabetes mellitus show a worldwide increase depending on the population [1,2]. In order to maintain normoglycemia with the development of insulin resistance (IR), a body of GDM pregnant woman secrete an increased amount of insulin, which is accompanied by less glucose utilization. Despite increased production, there is a relative insulin deficiency, and, as a consequence, hyperglycemia develops [3].

These conditions can lead to the harmful consequences, both for the mother and her offspring. GDM increases the chances of developing GDM again at the next pregnancies [4], it also increases the chances of developing type 2 diabetes at a later time with new-borns and mothers [5]. In addition to type 2 diabetes, the increased risk of development of metabolic disorders, and the obesity are observed. The major factors increasing the risk of GDM include obesity and a family history of diabetes, but also a sedentary lifestyle, age, polycystic ovary syndrome (PCOS), ethnics origin and exposure to toxicity [6].

Despite many studies on molecular biomarkers as early predictors of gestational diabetes, the oral glucose tolerance test (OGTT) is currently the preferred diagnostic method as the fastest and considerably inexpensive way to diagnose GDM.

GDM is usually diagnosed between 24 and 28 weeks of pregnancy. According to WHO, GDM should be diagnosed at any time in pregnancy if one or more of the following criteria are met: fasting plasma glucose ≥ 92 mg/dL (5.1 mmol/L) or ≥153 mg/dL (8.5 mmol/L) following a 75 g oral glucose load [7].

Apart from the OGTT, there are also other parameters which are examined during GDM diagnostics, e.g., HbA1c, which is not fully normalized because there is no particular established cut-off point. Otherwise, HbA1c ≥ 6.5% (48 mmol/mol) value is an indication of diabetes in general, and at any time during pregnancy. Non-pregnant women with an HbA1c between 5.7 and 6.4% (39–46 mmol/mol) are described as pre-diabetes. However, for the state of pregnancy, there is no specific range. Hughes et al. have pointed out the potential usefulness of an early HbA1c test. They showed that HbA1c value ≥ 5.9% (41 mmol/mol) is a potential marker for adverse pregnancy results and also for identifying women with pre-existing and undiagnosed diabetes in early pregnancy [8,9]. However, other physiological changes during pregnancy, of a different origin than diabetes, lower HbA1c levels and this may be the reason for the lack of a specific value range for HbA1c.

Fasting plasma glucose index (FPG) as a biomarker for GDM detection is also unreliable. Before the 24th week of pregnancy, the available data are insufficient to propose a new classification method or to determine a new diagnostic cut-off value. Corrado et al. [10] and Zhu et al. [11] undermined current opinions about the FPG index, exposing weakness in GDM diagnosis between 24 and 28 weeks of pregnancy, although they suggested considering the FPG value with the range given earlier as a high-risk marker. Cosson et al. [12], based on scientific literature and calculations and using a special algorithm, have shown that half of the patients from reviewed studies with an FPG value greater than 5.1 mmol/L meet the criteria for later onset of GDM.

Due to the increased risk of developing T2DM and cardiovascular diseases among women with GDM, and the negative impact of the GDM on offspring with an increased risk of metabolic diseases in the adult life, it is vital to understand pathogenesis and the molecular aspects of GDM to diagnose and treat GDM at the initial stage correctly.

The molecular aspects of GDM, in particular, transcriptomics and metabolomics approaches have been the focus of our research for several years [13,14,15]. These studies aim the elucidate the pathogenesis of GDM and postpartum developing of T2DM and the selection of potential biomarkers.

The metabolomic approach is focused on changes in metabolite profiles that indicate specific cellular processes. Translation of metabolomics knowledge into clinical applications may be promising in terms of identifying novel biomarkers and mechanisms related to diabetes mellitus.

Recently using the metabolomics approach we documented for the first time changed lipids profile of erythrocyte membranes in GDM women in comparison to normoglycemic (NGT) women, reporting the statistically significant changes in cis-vaccenic and stearic acids content [16].

Earlier metabolomic studies indicated that chain amino acids (AAs), particularly the branched ones, such as valine, leucine and isoleucine, as well as aromatic amino acids, tyrosine and phenylalanine could be used as markers for T2DM prediction [17].

Based on analysis of plasma and urine samples of pregnant GDM and normoglycemic women, Leitner et al. [18] indicated tryptophan as a substantial variable differentiating these two groups. Since tryptophan is biotransformed to serotonin, it has been hypothesized that serotonin metabolism may also change in GDM. They revealed that serotonin and the related metabolites differ significantly between the control patients and GDMs, which could confirm the contribution of serotonin metabolism to GDM. On the other hand, in 2019 López-Hernández et al. [19] identified metabolites belonging to following classes of compounds: benzopyrans, carboxylic acids and derivatives, glycerolipids, indoles and their derivatives, tetrapyrroles, sphingolipids, and steroid derivatives, that were significantly elevated in the urine of patients with GDM suggesting biochemical and metabolic changes in GDM that may be associated with steroid hormone biosynthesis pathways and tryptophan metabolism (TRP) pathways. In another study, Bentley-Lewis et al. [20] found that levels of the six following metabolites: alanine, glutamate, serine as well as anthranilic acid, allantoin, creatinine, differed between the GDM and control groups.

One of the amino acids, which has a considerable impact during pregnancy, is L-arginine. This is one of the essential amino acid and its deficiency may lead to pregnancy complications such as preeclampsia [21], hypertensive, intrauterine growth restriction (IUGR) [22] and embryonic loss [23]. Moreover, L-arginine is responsible for the regulation of numerous pathways vital to reproduction, growth, and health.

Besides, L-arginine is a substrate for the synthesis of nitric oxide (NO). This small inorganic molecule plays an essential role in steroidogenesis, oviduct functions, ovulation, embryo implantation, maintenance of pregnancy and the initiation of labor [24,25,26]. NO is also one of the most critical vascular signaling molecules which relaxes and widens blood vessels and reduces platelet sensitivity to pro-aggregating agents.

During pregnancy, the biosynthesis of NO is increased, which is related to the maternal vasodilatation associated with gestation and can lead to maternal L-arginine deficiency during pregnancy due to consumption by the fetus [27,28].

There has been increasing attention paid to the role of L-arginine to promote insulin secretion and improve insulin sensitivity [29,30]. What is more, L-arginine also plays a role in the treatment and prevention of cardiovascular and cerebrovascular diseases [31,32].

Thus, the present studies have been conducted to examine the follow-up amino acids profiling of women diagnosed with GDM during pregnancy, three months and one year after delivery.

Identification of biomarkers like AAs, obtained from blood in early pregnancy, three months and one year after delivery, may contribute to complementation of existing clinical risk factors to identify women at high risk of developing GDM.

## 2. Results

During pregnancy, significantly higher values of fasting plasma glucose (FPG), 2h OGGT, insulin, and homeostasis model assessment of insulin resistance (HOMA-IR) occurred in GDM patients in comparison to control, as was expected (Table 1). No statistical differences between GDM and NGT were noticed in terms of HbA1C, CRP, HOMA-B, CH, LDL, HDL and TG as well as age, gestational weight, and pre-pregnancy BMI. The most significant differences were observed for insulin (1.51), and HOMA-IR (1.62), which were respectively larger, while in the fasting plasma glucose and 2h OGGT test, the differences were 1.06 and 1.11, respectively.

**Figure 1:**
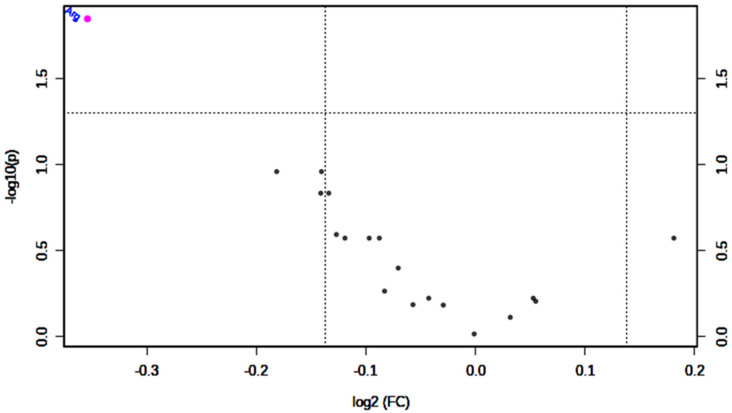
Important features selected by volcano plot with fold change threshold (x) 1.1 and U Mann–Whitney’s test threshold (y) 0.05. The red circle represents features above the threshold. Both fold changes and *p* values are log transformed.

**Figure 2:**
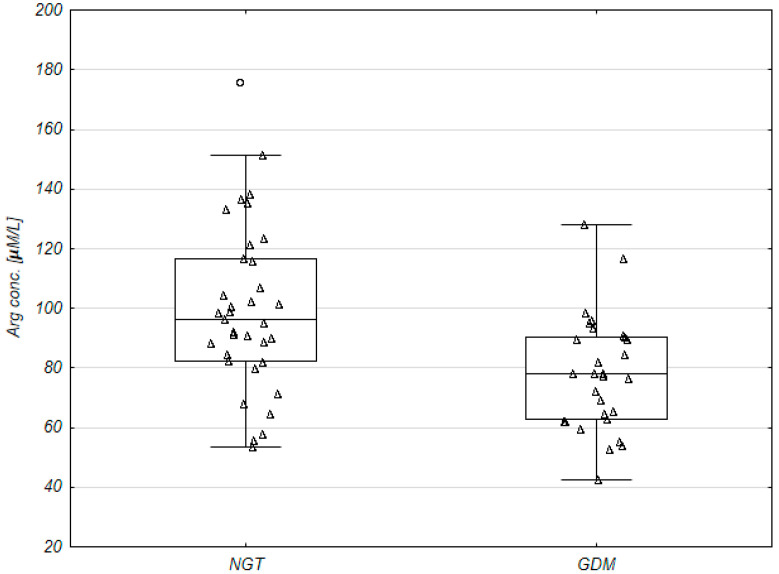
Arginine concentration in plasma of pregnant women with normal glucose tolerance (NGT; *n* = 35) and gestational diabetes mellitus (GDM; *n* = 29). Row data shown as triangular points. Boxes represent the median and interquartile range (25–75%), whiskers—non-outlier range, and circles—outliers.

A targeted metabolomic analysis was performed to evaluate changes in the concentration of amino acids in plasma of GDM and NGT women (Table 2). In the GDM group, the following amino acids: Arg, Gln, His, Met, Phe, Ser were decreased with statistical significance in comparison to the NGT (U Mann–Whitney test). Among amino acids for Arg surprisingly most significant decrease was observed in GDM versus NGT (the 0.81-fold difference between the groups; *p* = 0.001; Table 2, Figure 1 and Figure 2).

**Figure 3:**
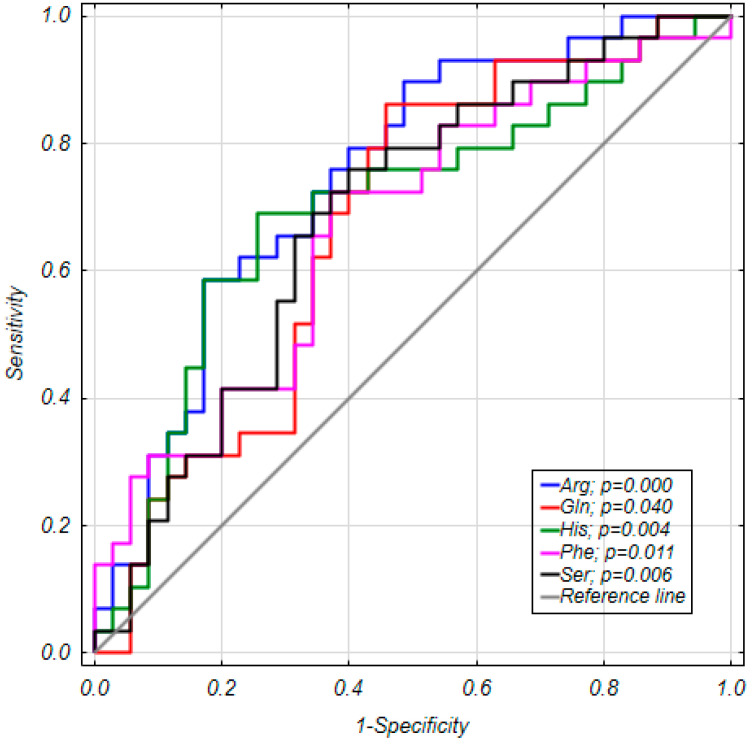
ROC curves for five most relevant amino acids that possess some NGT-GDM discriminative potential. *p*-values for null hypothesis (H0) AUC *=* 0.5.

By analyzing the association of all amino acid concentration results of GDM and NGT women from pregnancy on impaired glucose tolerance (IGT), the statistical models predicting diabetic status were created with an arginine-based model to be the most interesting of the obtained models. The model detected one variable characteristic in GDM-arginine (AUC = 0.749, Table 2, Figure 3), which was statistically significant and this model could predict GDM. To assess the correctness (sensitivity and specificity) of selected predictors in the created model, a receiver operating characteristic (ROC) curve was prepared (Table 2, Figure 3).

In order to show differences between GDM, and NGT groups, Volcano plot of log2 (fold change: (GDM/NGT) versus –log10 (Mann–Whitney *p*-value) was prepared (Figure 1). The red circle points on Arg, which represents features above the threshold and represent values that display high statistical significance in distinguishing GDM from NGT.

**Figure 4:**
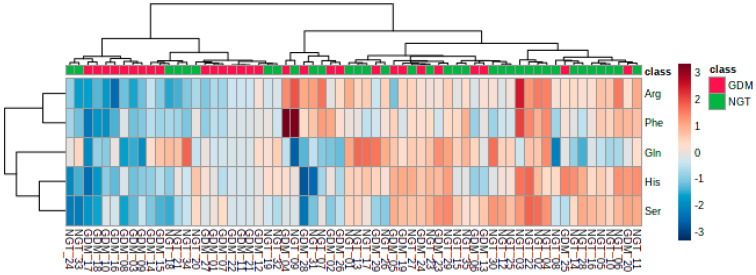
Clustering result is shown as heatmap (distance measure using Euclidean, and clustering algorithm using Ward’s method) for five most significant amino acids. Data are log-transformed and standardized.

The distribution of the most altered amino acids in GDM versus NGT was further analyzed using hierarchical cluster analysis (HCA) using Ward’s method, which highlights the similarity between different cultivars based on the amino acids they contain. The computed HCA plot in Figure 4 indicates that the samples were separated into several groups with the most tolerant cultivars clustering together.

### PCA Analysis

**Figure 5:**
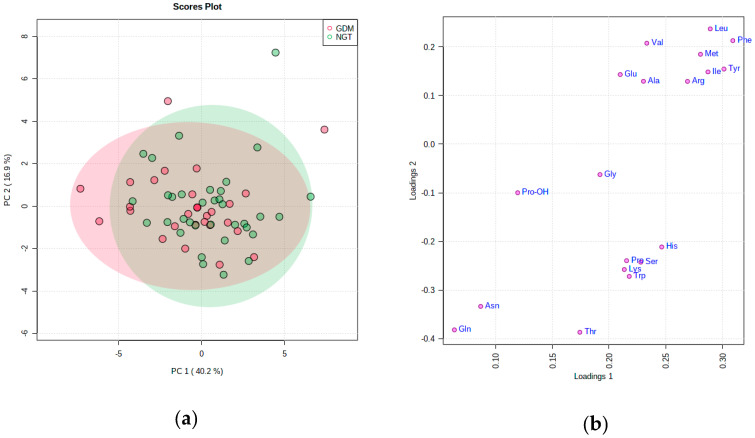
Principal component analysis (PCA) results for amino acids concentrations in pregnant NGT and GDM patients group. (**a**) Scores plot between the PC1 and PC2 for NGT and GDM patients. The explained variances are shown in brackets; (**b**) loadings plot of 19 amino acids for PC1 (Loadings 1) and PC2 (Loadings 2).

To provide comparative interpretations and visualization of the metabolic amino acids changes in this study, a principal component analysis (PCA) was applied to the UPLC-MS spectral datasets (Figures S1 and S2). The first two principal components (PC1 and PC2) explained 57% of overall data variance, but failed to resolve the NGT-GDM identity. No other PCs combination provided a significant group discrimination, leading to the conclusion that the simplest single-variable models were the best available from the dataset. The component’s loading coefficients of all the raw amino acid variables showed some structure with distinct grouping in the context of PC1 (Loading 1) and PC2 (Loading 2) dimension which is illustrated on the PCA loadings plot (Figure 5b).

**Figure 6:**
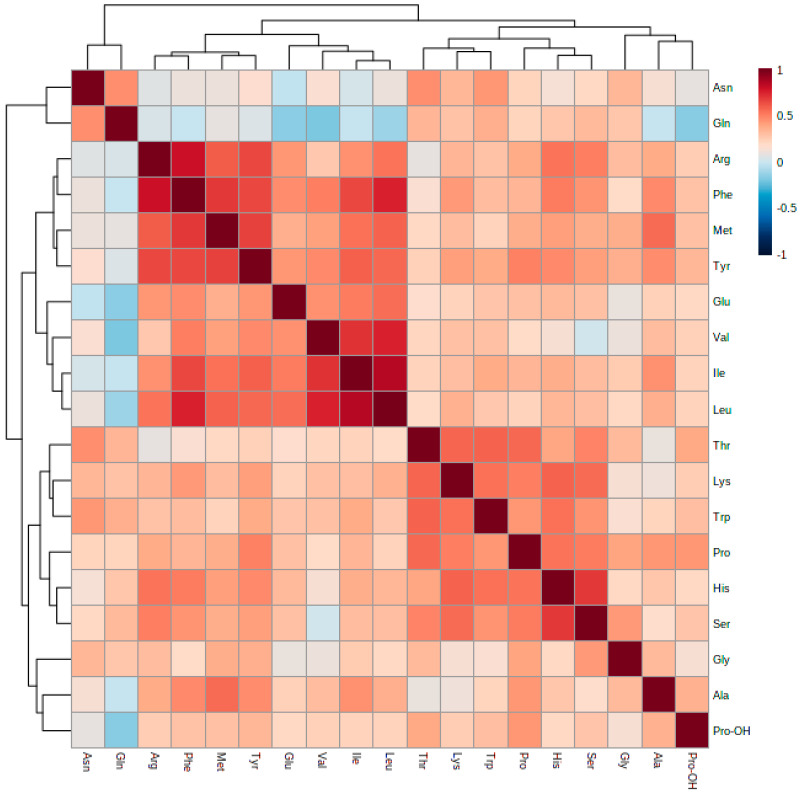
Overall Spearman’s correlation heatmap for studied amino acids in pregnant NGT and GDM women (*n =* 64).

In order to find relations between amino acids, a correlations heatmap was prepared. The strongest positive correlations for the following amino acids: His, Ser, Ile as well as for Leu and Val were identified (Figure 6).

**Figure 7:**
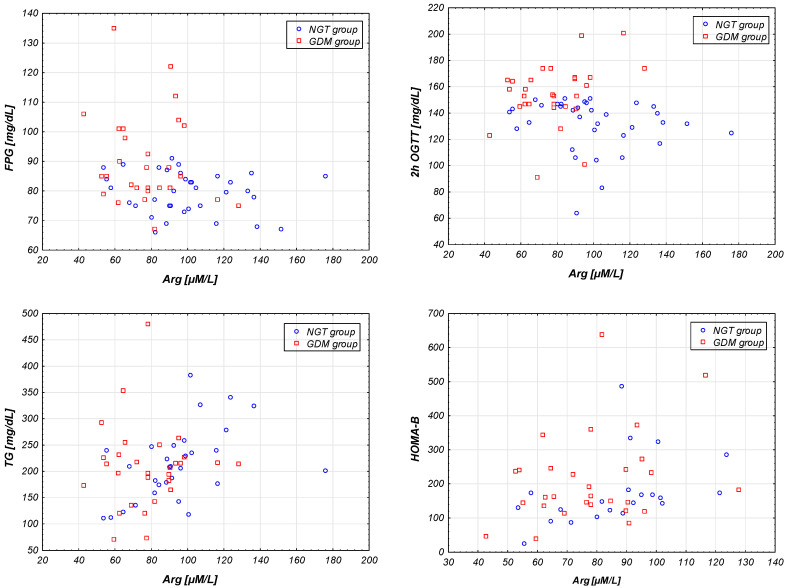
Scatter plots imaging the strongest correlations between arginine concentration and biochemical characteristics (FPG, OGTT 120, TG, HOMA-B) of studied pregnant NGT and GDM women (*n =* 64).

We performed a correlation analysis between arginine plasma concentration (µM/L) and anthropometric and biochemical characteristics (including amino acids) in the entire studied group of patients during pregnancy as was presented in Table 3. A medium positive correlation of Arg with TG and HOMA-B was observed. The strongest negative association of Arg with FPG, 2h OGTT, were observed (Figure 7) but after Benjamini–Hochberg correction they were not statistically significant.

A HPLC–MS-based approach was applied for plasma amino acids analysis of control and GDM women at 24–28 week of gestation, as well as three months and one year postpartum. With the Friedman’s ANOVA test, we analyzed time-related changes in AA concentrations in the GDM group (Table 4). The analysis of 18 amino acids revealed significant differences in amino acid profiles in plasma during pregnancy, 3 and 12 months after delivery. Surprisingly, among the eighteen amino acids analyzed, only Arg did not show a significant change during the whole period of the study (Table 4). Two of all studied amino acids, i.e., tryptophan, and serine, showed an upward trend in both time intervals. The second compartment, covering initially upward in the first study interval (3Mo/GDM) and then unchanged trends, contains thirteen amino acids, Ala, Glu, Gln, Gly, Ile, Leu, Lys, Met, Phe, Pro, trans-4-hydroxy-L-proline (Hyp), Tyr, and Val. Two amino acids, namely Thr, and His initially decreased, then were kept at the same level. The metabolic characteristic provided for the studied group, including, 2h OGTT, insulin, TG, TC, HDL-C, LDL-C, and CRP, initially decreased and then were unchanged. Metabolic parameters encompassed HOMA-B, and HOMA-IR, were unchanged during the study time. All results of the variables in Friedman’s ANOVA test except arginine and HbA1C showed statistically significant changes.

Wilcoxon test analysis of amino acids and biochemical parameters profile in GDM versus three months after delivery revealed that twelve amino acids and seven biochemical factors differed significantly. Among the tested amino acids, Ala, Arg, Gln, Gly, Glu, Ile, Leu, Met, Ser, Trp, Tyr (with fold differences 1.25; 1.09; 1.36; 2.08; 1.20; 1.19; 1.29; 1.16; 1.07; 1.11; 1.58, respectively) showed a significant increase after three months after delivery, whereas His, and Thr significantly decreased (with fold changes 0.91 and 0.59, respectively) The biochemical changes in 2h-OGGT, insulin, TG, TC, HDL-C, LDL-C, CRP (fold changes 0.62, 0.65, 0.38, 0.77; 0.81, 0.91, and 0.37, respectively) revealed a decrease, while for FPG, an increase was observed but was not statistically significant at this time point. After one year, significant differences were shown by fifteen amino acids and seven biochemical factors. The following amino acids: Asn, Gln, Glu, Gly, Ile, Leu, Lys, Met, Phe, Pro, Ser. Trp, Tyr, Val (with fold differences 1.18, 1.43, 1.14, 1.78, 1.20, 1.28, 1.27, 1.12, 1.49, 1.36; 1.27, 1.59, and 1.54, respectively) exhibited statistical increase versus NGT. An opposite trend was observed for threonine with statistically significant decrease (with fold change 0.77). Among biochemical variables, 2h-OGTT, insulin, TG, TC, HDL-C, LDL-C, CRP (with fold changes 0.66, 0.77, 0.42, 0.75, 0.84, 0.75, and 0.29, respectively) showed a significant decrease one year after birth, except for fasting insulin and FPG, which were relatively higher than GDM without statistical significance.

The differences in amino acid concentrations between 3M and NGT and 1Y and NGT with the use of Mann–Whitney U analysis were also analyzed (Table 5). In the comparison of three months postpartum results to the control group, twelve amino acids as follows: Ala, Gln, Glu, Gly, His, Leu, Pro, Pro-OH, Thr, Trp, Tyr, and Val differed with statistical significance. Among these amino acids, only Thr was significantly decreased after one year, significant differences were observed for twelve amino acids. On the one hand, ten amino acids showed an average increase, and on the other hand, a significant decrease was recorded for threonine and arginine.

Since that, in our experiments, we did not have control samples for 3M and 1Y study groups, we compared the results 1Y after pregnancy with the data available in the Human Metabolome Database (HMDB Version 4.0, www.hmdb.ca) with the use of Student’s t test for normal healthy women (Table 6).

A comparison of the patients’ amino acid concentrations one year after delivery with data from the HMDB database revealed that only three amino acids, including Arg, Ile, and Ser, had comparable values with the data from HMDB. Unexpectedly, for Gln, and Glu, about twofold increase was observed (with fold changes of 2.29 and 1.98, respectively; Table 6).

## 3. Discussion

Irrespective of several recommendations, there is no standardized methodology for diagnosing GDM and the diagnostic criteria vary among countries and between the recommending organizations [34]. It is known that concomitant hyperglycemia may trigger metabolic alterations that profoundly affect energy metabolism across the whole body including pancreas, muscle, adipose tissue, and liver. Metabolomics is a useful tool with the potential to determine the set of metabolites that may be promising in the prediction of gestational diabetes mellitus. It is well known that amino acid levels are distinctly perturbed in patients with diabetes, therefore identifying predictive biomarkers in order to avoid chronic abnormalities in metabolism, in both mother and fetus, is a considerable challenge.

In the present study, we examined the amino acids profiles in GDM as well as three months and one year postpartum. The biochemical and anthropometric parameters during pregnancy revealed statistically significantly higher values of FPG, 2h OGGT, insulin, and HOMA-IR in GDM patients against the control (Table 1). In T2DM and GDM, increased insulin response, decreased insulin sensitivity and suppressed hepatic glucose production during insulin infusion are detected [35]. In our study, HOMA-IR statistically significantly increased in GDM, which points to high IR. The high levels of HOMA-IR and HOMA-B and fasting insulin in GDM women indicate IR and intense beta cell load. Earlier, Wei Bao et al. showed a significant relationship between the occurrence of GDM and increased plasma TG and lower HDL-C [36], which was also observed in our research, but in our case the changes were not statistically significant.

The analysis of amino acids concentrations in GDM women revealed downregulation in Arg, Gln, His, Met, Phe, Ser with the most substantial decrease in Arg and those metabolites seem to be significantly associated with GDM and glucose-mediated pathways. For example, glutamine is associated with the development of hyperglycemia [37]. Research findings have confirmed that branched-chain amino acids (BCAAs), including isoleucine, leucine, and valines as well as aromatic amino acids such as phenylalanine and tyrosine are involved in pathways of insulin resistance, including fatty acid oxidation, mTOR, JNK and IRS1 pathways and also T2D [38]. However, there are many discrepancies about the association between BCAAs and GDM. In line with our observation that BCAA are not changed in GDM except only valine which not significantly increased in GDM versus NGT, some authors reported that serum or fasting urine samples obtained during the second and third trimester of pregnancy did not report any significant changes in BCAA levels [39,40,41]. The elevated valine levels in the GDM group can thus contribute to decreased insulin signaling and worsen insulin resistance. In contrast to our results, Pappa et al. and How et al. observed in their studies that BCAA were elevated in GDM, versus normoglycemic women [42,43]. It is worth noting, however, that when comparing the population in the studies, differences in the age and BMI of patients with GDM should be taken into account. In the study of Pappa, women with GDM had an increased BMI contrary to our patients and those from the Hou study. In terms of age, all patients were in a similar peer group. This can clarify the inconsistent reports on BCAA levels in GDM. In earlier studies, the targeted metabolomics analyses have demonstrated the increased level of alanine, proline, glutamine/glutamate, arginine, and asparagine/aspartate in subjects with a higher level of fasting blood sugar [44].

Numerous studies indicate that in conditions of insulin-resistance and diabetes, the level of circulating methionine and its catabolic derivative cysteine is increased [45,46]. In our study in GDM women, methionine concentration decreased. A similar correlation was also confirmed by Pappa et al. who, among 21 analyzed amino acids, found that only methionine, glycine, alanine, citrulline, and ornithine levels were significantly higher in normal pregnant women compared to those with GDM [42]. However, it is worth mentioning is the difference between the several of the earlier studies and our report in the context of BMI, where our patients were not obese and the BMI shows no statistically significant changes. Metabolism of methionine occurs mainly in the liver with participation of methionine adenosyl transferase which catalyzes methionine into S-adenosylmethionine—the methyl donor for DNA or protein methylation [47]. The process of DNA methylation, in turn, is connected with glucose metabolism, and insulin resistance. Moreover, it is also involved in β cell dysfunction—that is why altered or abnormal DNA methylation ultimately leads to the pathogenesis of diabetes [48].

The aromatic amino acids, including phenylalanine, are involved in the etiology of liver disorders [49]. Our results display that women with diagnosed GDM have a lower concentration of Phe compared to the NGT group. The studies of other authors do not confirm our observation. Butte et al. [50] reported the higher level of fasting and postprandial aromatic amino acids in the plasma of GDM women at 32–36 weeks of gestation. On the other hand, Metzger et al. [51] and Cetin et al. [39] observed no changes in phenylalanine levels in maternal plasma.

Moreover, using the advanced statistical tools we applied models that differentiated among individuals with GDM and, by diverse combinations of amino acids, the model comprising Arg turned out to be the optimal one (AUC = 0.749; 95% CI, 0.96 (0.934–0.986); *p* < 0.001). By analyzing this model, it can be concluded that arginine may play a crucial role in the pathogenesis of GDM and could be a good biomarker in earlier detection of this disease. In 2018 Roy et al. presented a model based on five metabolites (Leu, Glu, carnitine metabolites acethylcarntine-C2, butyryl-carnitine-C4 and isobutyryl-carnitine-IsoC4), associated with a significant increase in GDM risk. However, this study had some limitations. It was conducted in a clinical setting in the first trimester, hence, women who attended their first prenatal clinical visit were non-fasting. In this study, a decrease in Glu concentration reduces the chance of GDM, and points at the inverse relationship between Glu and its derivatives in metabolic pathways, in the development of GDM in a different manner. It is known, that data from non-fasting patients may show bigger differentiation of the results [52]. Our studies were based on data from pregnancy between 24 and 28 weeks and finally the proposed model contains only one variable—arginine. Furthermore, patients enrolled in our study were tested in fasting state, so it can be said that the results are not subject to underestimation and variance [52]. Our results are also in line with the study by Chorell et al. that demonstrated, on the one hand, significant postpartum increase in alanine and arginine in both the NGT-risk and the GDM groups, and, on the other hand, increase in leucine and proline only in the GDM group. The explanation for this process may be related to the fact that these amino acids stimulate insulin secretion and could, thereby, contribute to exhaustion of β-cells by causing endoplasmic reticulum stress [53]. In our work, only Arg was also decreased and for Ala opposite trend was observed. The comparison our population with those in Chorell study revealed that our GDM patients were not obese.

As opposed to our results, a study Rahimi et al. found a significantly elevated plasma level of asparagine, threonine, aspartate, phenylalanine, glutamate, and arginine in GDM women after adjustment for gestational age and BMI [46]. Nevertheless, some results are available for GDM in the study of Piatti et al., who demonstrated that Arg significantly improves insulin sensitivity in T2DM, but does not completely normalize peripheral and hepatic insulin sensitivity in type 2 diabetic patients [54].

**Figure 8:**
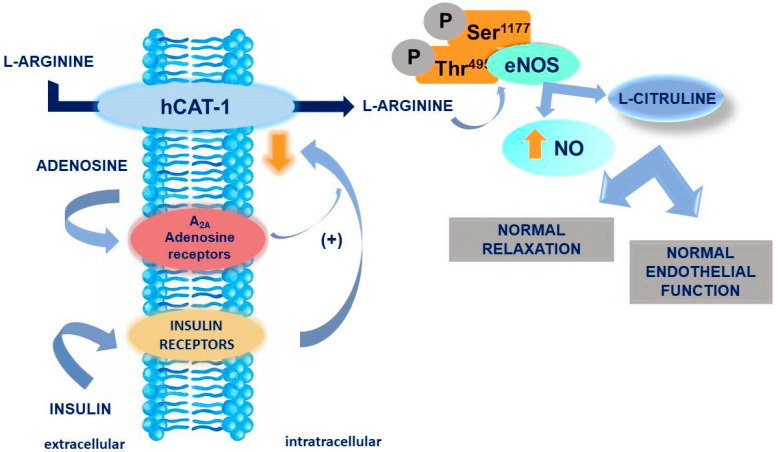
The role of L-arginine transport mediated by human cationic amino acid transporters 1 (hCAT-1) in nitric oxide (NO) and L-citrulline formation via endothelial NO synthase (eNOS). eNOS activity results from a balanced degree of phosphorylation of threonine 495 (P-Thr495) and serine 1177 (P-Ser1177).

The effects of L-arginine in diabetes could be mediated by NO as well (Figure 8). However, the results on the role of NO in diabetes are contradictory as was reported in a rat model [55]. Shengdi Hu et al., showed that arginine, which is a precursor of NO, can lead to T2DM retardation through a mechanism including modulating glucose homeostasis and increasing insulin sensitivity. Moreover, arginine has the potential to stimulate beta-cell neogenesis, increasing the area of insulin-positive cells. Among other studies, a potential relationship between IR and endothelial response to NO synthesis inhibition can be found, because of the lack of arginine [56]. Furthermore, arginine as a precursor of NO can increase vasodilation through NO among people with high IR. The role of arginine in GDM may be linked to increased activity of the ALANO pathway in GDM that involves extracellular adenosine accumulation resulting from reduced of adenosine uptake into endothelial cells [57,58]. The previous study of our group has shown that maternal leukocyte ADORA2B overexpression is associated with hyperglycemia in GDM subjects, and complex alteration accompany it in the expression of diabetes-related genes involved in insulin action, carbohydrate and lipid metabolism, oxidative stress, and inflammation [13]. Another study showed a potential association between reduced NO and increased insulin resistance (IR) [59]. Other studies have additionally demonstrated the involvement of phosphatidylinositide 3-kinases (PI3K) in the arginine NO (AMPK) pathway, in which the supply of more arginine leads to an increase in NO levels which up-regulate PI3K and, ultimately, improves muscle and liver insulin sensitivity [56,60,61]. However, the effects of arginine can also be opposite as was revealed in vitro studies which showed that NO has cytotoxic properties destroying pancreatic β cells, whereas NOS inhibitors may act as protectors [62,63]. Our research results have shed light on the role of arginine in the development of GDM It is worth mentioning that even within a homogeneous population which is the GDM group, the molecular characteristic could be heterogeneous. Taking into account our results regarding overexpression of SIRT1 in GDM women, we demonstrated that there were women with increased level of SIRT1 but, on the other hand, there were also women with not significantly changed level of SIRT1 within this group. It is worth mentioning that even within a homogeneous population which is the GDM group, the molecular characteristic could be heterogeneous. Taking into account our results regarding overexpression of SIRT1 in GDM women, we demonstrated that there were women with increased level of SIRT1 but, on the other hand, there were also women with not significantly changed level of SIRT1 within this group.

Multivariate PCA models limited exclusively to all studied women showed weak separation between those that were normoglycemic and those with recognized gestational diabetes mellitus. Notably, it is visible that not all of the significantly changed amino acids clustered together with only Phe and Met grouping loosely with Arg. To a degree, there also emerged clustering inside the amino acids types, i.e., branched, alkaline and aromatic amino acids groups. It can be seen in PCA modeling that certain groups of amino acids occur together indicating their similar pattern of concentration within studied group.

Additionally, based on Spearman’s rank correlation of all amino acids, we investigated the correlations of Arg with biochemical and metabolic factors. The strongest negative correlations between arginine concentration and biochemical characteristics FPG, 2h OGTT, and positive for TG, HOMA-B of studied pregnant NGT and GDM women were observed but after Benjamini–Hochberg correction, they were not statistically significant.

The scope of correlation indicates a moderately strong relationship between Arg and related factors, indicating a protective effect of arginine on the subsequent occurrence of diabetes. The negative correlation between asparagine and TGs may indicate participation in lowering the level of TGs, a source of free fatty acids, inhibitors of the insulin pathway.

As this longitudinal follow-up study provided information of glycemic condition after delivery, we explored whether the amino acids profile was different in women diagnosed with GDM, as compared to those with normal glucose tolerance three months and one year postpartum.

According to Friedman’s ANOVA test, almost all the factors tested showed statistically significant differences during the whole test, except arginine and HbA1c, which means the values of the variables differed between measurements during the study.

Analysis with the use of the Wilcoxon signed-rank test mostly confirms the results of differences for the studied variables from Friedman’s test. The test showed the most variable differences between the time of pregnancy and three months postpartum, and the least significant differences between 3 and 12 months postpartum. The analysis of clinical data three months after delivery showed a significant decrease in 2h OGTT, insulin, TG, and total cholesterol, HDL-C, LDL-C, CRP (Table 2). Although after three months, the insulin was still lower compared to pregnancy, a lesser extent, these results may indicate a normalization of factors which suggests recovery of GDM. Some of the women could have some degree of glucose intolerance after delivery.

During pregnancy, the body requires adaptation by placental and nonplacental hormones, which prepares the body for metabolic stress associated with fetal development [64]. Restoration of maternal insulin sensitivity after delivery is a necessary physiological and metabolic adaptation during female reproductive life and, according to John P. Kirwan et al., it lasts about one year, where insulin sensitivity improves by 74% from late pregnancy [65].

We also found significant three months postpartum increase in concentration of some amino acids (Table 4) with concomitant significantly decrease in concentration of His and Thr similarly with previous research findings [53]. These results suggest that increased levels of BCAA or other mitotoxic/lipotoxic metabolites from these amino acids might increase the risk for T2DM through their contribution to the development of insulin resistance. An increase in alanine, glutamic acid concentration, in GDM cases may indicate an enhanced gluconeogenesis process, but in our study this change was not statistically significant.

The postpartum increase may reflect disturbances in glucose metabolism after GDM and may be linked with an increased risk of postpartum T2DM [17].

By analyzing the biochemical data and amino acids concentrations one year after delivery, it can be stated that some amino acids were significantly increased versus NGT, suggesting metabolic transformations that may be observed in GDM (Table 4). An opposite trend was observed for threonine with a statistically significant decrease. Among biochemical variables, 2h-OGTT, insulin, TG-, TC, HDL-C, LDL-C, CRP showed a significant decrease one year after birth, except for fasting insulin and FPG, which were relatively higher than GDM without statistical significance. Since the variation in Arg concentration during pregnancy and postpartum is not statistically significant, it cannot unambiguously determine the participation of Arg in the normalization processes after pregnancy. The results obtained in the study mostly stay in line with the results obtained by other researchers, although the majority of significant differences concern the period after pregnancy rather than in the pregnancy itself. The consistent results are related to leucine, isoleucine, valine, asparagine, tyrosine, glutamic acid, tryptophan, methionine, glycine, alanine, while arginine, proline and histidine showed significantly contradictory results compared to other studies performed. In the results of the current study, it must take into account that, the GDM women were not compared to the controls three and twelve months postpartum because no data for NGT women from this period are available. We tried to assess the influence of the pregnancy on metabolic transition profile in the GDM women by comparing postpartum results with data from the HMDB database, and we can state that only three amino acids, including Arg, Ile, and Phe, had comparable values with the data from HMDB. Unexpectedly, for Gln, and Glu, about a twofold increase was observed (with fold change 2.29, and serum metabolomics analysis of women at late pregnancy; therefore, the evolutionary process was 1.98, respectively; Table 6). These results suggest that during pregnancy, alteration in amino acids profile may take place.

Our study had some limitations that should be taken into consideration. In our study, the analysis of metabolites variances could not be assessed throughout pregnancy. Large cohorts, dynamic monitoring of metabolites during pregnancy, comparing the GDM and postpartum sample to the well-designed controls can improve our understanding of metabolites alteration and verify the validity of models of GDM. Dissimilarities among findings from the studies could be caused by ethnic origin and size of the study populations, differences in GDM diagnostic criteria, specimen prepared for test and metabolites profiling platforms, and the timing of metabolome profiling. It is also worth noting that our GDM patients were not obese.

Therefore, the mechanisms related to gestational diabetes require further explanation. Future investigations that follow the same strict guidelines are needed to improve the replication of findings.

## 4. Materials and Methods

### 4.1. Study Population

All participants were recruited from Diabetes Clinic “Bluemedica” (Lodz, Poland) and Department of Internal Medicine and Diabetology of the Medical University of Lodz. The GDM was diagnosed according to WHO diagnostic criteria [1] as fasting glucose level ≥92 mg/dL (5.1 mmol/L) or ≥153 mg/dL (8.5 mmol/L) after 2-h 75-g OGTT. After diagnosis, the control group included 35 healthy pregnant women with normal glucose tolerance (NGT) and GDM group included 29 women with impaired glycemia (64 patients in total). Fasting blood samples were collected into EDTA containing tubes at 24–28 weeks of gestation (at the day of the OGTT) for both the NGT and GDM group. For the GDM group, samples were also collected three months and one year after delivery, so they were examined at the three different times in a follow-up manner. The inclusion criteria are shown in Table 7.

The study was approved by Bioethics Committee of the Medical University of Lodz (approval code: KE/963/18 dated 10 July 2018).

### 4.2. Anthropometric Measurements and Biochemical Data

Maternal weight and height in the third trimester of pregnancy were measured using standard equipment, which allowed us to calculate weight gain and pre-pregnancy body mass index (BMI).

Blood samples were taken from patients after 12 h of fasting. Utilizing an enzymatic colorimetric method using kits, the total cholesterol CHOD-PAP and triglyceride GPO-PAP (Roche Diagnostics GmbH, Mannheim, Germany), serum triglyceride (TG) levels as well as HDL and LDL cholesterol were measured [66,67]. Glycated hemoglobin (HbA1C) was measured using a latex agglutination test, a turbidimetric immunoassay [68]. C-reactive protein (CRP) concentration was determined, according to the manufacturer’s instructions, with the use of the cassette COBAS INTEGRA CRP (Latex) in a turbidimetric manner (Roche Diagnostics GmbH, Mannheim, Germany) [69]. Elecsys insulin assay was used to determine the concentration of plasma insulin (Roche Diagnostics GmbH, Mannheim, Germany) [70].

For each patient, an indicator of insulin resistance (HOMA-IR) and beta-cell function (HOMA-B) was calculated with the use of the homeostasis model assessment (HOMA) as follows [71]:

(1) $$\mathrm{HOMA}-\mathrm{IR}\;=\;\;\mathrm{fasting}\;\mathrm{insulin}\;\left(\frac{\mu U}{\mathrm{mL}}\right)*\mathrm{fasting}\;\mathrm{glucose}(\frac{\mathrm{mg}}{\mathrm{dL}})/405;$$

(2) $$\mathrm{HOMA}\;B\;=\;360\;*\;\mathrm{fasting}\;\mathrm{insulin}\left(\frac{\mu U}{\mathrm{mL}}\right)/\left(\mathrm{fasting}\;\mathrm{glucose}\left(\frac{\mathrm{mg}}{\mathrm{dL}}\right)-63\right)$$

### 4.3. Chemicals and Reagents

The methanol used in the procedure was from Sigma Aldrich (Sigma Aldrich Chemie GmbH, Steinheim, Germany). A solution of internal, deuterated standards for amino acids concentration calculation, was produced by CIL (Cambridge Isotope Laboratories, Andover, MA, USA). For derivatization by butylation of the carboxyl group of the analyte and formation of the butyl ester, 3N HCl in *n*-butanol (3N Hydrochloric Acid in 1-Butanol) was used from REGIS TECHNOLOGIES, INC. (Austin Avenue, Morton Grove, IL 60053 USA). Formic acid (FA), acetonitrile (ACN), heptafluorobutyric acid (HFBA) were ordered from J. T. Baker (Avantor Performance Materials B.V., Deventer, Holand).

### 4.4. Sample Preparation

A volume of 10 µL of patient plasma with 10 µL internal standard solution was placed in a polypropylene deep-well plate. Precipitation and extraction were performed by adding 780 µL of 1N HCl in methanol. An internal standard solution contains 125 nmol/mL Gly labelled and 25 nmol/mL labelled Ala, Asp, Glu, Leu, Met, Phe, Tyr, Val, Orn, Cit. Samples were mixed on an orbital shaker at 600 rpm, for 10 min, at RT. Precipitation was additionally improved by incubation at −20 °C for 2 h. Samples were centrifuged at 1000 rpm for 5 min. Subsequently, 50 µL of supernatant was transferred to a new 96-well plate with (250 µL total well volume). The contents of the well plate were evaporated to dryness for about 15 min; 25 µL 3N HCl in *n*-butanol was added to each well. The samples prepared in this way were incubated at 60 °C for 25 min and dried for about 15 min. The residue was dissolved in 200 µL H2O: MeOH (80:20) + 0.1% FA. The dissolved samples were mixed on a shaker at 600 rpm for 10 min, at RT.

### 4.5. HPLC–MS Analysis

HPLC–MS/MS analyses were performed by ExionLC (AB Sciex) liquid chromatograph equipped with an Exigent autosampler (AB Sciex) coupled with tandem mass spectrometer (4500 QTRAP, AB Sciex) with electrospray ion source (Turbo V, AB Sciex). An amount of 2 µL of the dissolved sample was injected for analysis into ACE Excel C18 column with dimensions of 2.1 mm ×50 mm × 1.7 µm. Flow ratio of the column was 0.4 mL/min with a temperature of 40 °C. H_2_O with 0.5mM HFBA and MeOH in proportion with (1:1) ACN constituted, respectively, eluents A and B. The time and used a gradient of the eluents are following: O min. eluent A: 80%, eluent B: 20%; time 7 min. eluent A: 50%, eluent B: 50%; time 7.1–9 min. eluent A: 5%, eluent B: 95%; 9.1–11 min. eluent A: 80%, eluent B: 20%.

### 4.6. Statistical Analysis

Statistical analysis was performed using Statistica 13.1 together with the Plus set (StatSoft Polska Sp. z o.o., Poland). For some multidimensional analysis, i.e., clustering and PCA, a website platform MetaboAnalyst 4.0 (Sainte-Anne-de-Bellevue, Kanada, https://www.metaboanalyst.ca) was used [30]. Quantitative data were presented in the form of median together with the value of the upper and lower quartiles. In Table 5, data were shown as a mean with standard deviation. The differences in medians between study groups to the control group were expressed as fold change. Where relevant, information about the number of individuals or observations in the sample (*n*-value) was also included. Control and research data regarding anthropological and biochemical features were verified by the Shapiro–Wilk test to check the normality of the distribution. The Levene’s test and Bartlett’s test were used to verifying the homogeneity of variance in each subset of the data. Due to the failure to meet the parametric tests’ assumptions for many variables, the non-parametric alternative was performed, i.e., the Mann–Whitney U test for independent two groups (GDM vs. NGT) comparison. Simultaneously, Friedman’s ANOVA test for repeated measures in the follow-up group was used. The Wilcoxon signed-rank test was used to posthoc pairwise comparisons of the data between follow-up time points. Due to the lack of normoglycemic non-pregnant control group in our study, the HMDB records were used as an external reference for amino acids mean concentrations and the student’s t test was performed to check the differences between the means. Furthermore, the obtained biochemical and anthropometric characteristics of patients were subjected to Spearman’s rank correlation analysis. For a more detailed analysis of the association of amino acids concentration and GDM status, the univariate logistic regression analysis was performed, where odds ratios and Wald’s test and Likelihood Ratio (LR) test *p*-values were the output. Additionally, the receiver operating characteristic (ROC) curves were analyzed so as to indicate the most relevant NGT-GDM diagnostic amino acids.

In order to describe the amino acids’ concentration structure and relationship, the principal components analysis (PCA) was performed. To reach insight into the discriminative power of the amino acids NGT-GDM group, the approach of hierarchical clustering was conducted with distance measure using the Euclidean method, and the clustering algorithm using Ward’s method. Data were subjected to logarithmic transformation and standardization (auto-scaling) before clustering and PCA analysis (Supplementary).

In all analysis, *p* values equal to or less than 0.05 were considered to be statistically significant. For multiple hypothesis’ testing among variables within the same patients’ group, the Benjamini–Hochberg correction was applied.

## 5. Conclusions

In this study, we used metabolomics approach to find metabolites related to gestational diabetes mellitus, and postpartum changes characteristic for possible T2DM. Our targeted metabolomics analysis demonstrated decreased plasma concentration of Arg, Gln, His, Met, Phe, Ser in GDM women and, among them, Arg seems to be interesting. Moreover, we established models that differentiated among individuals with GDM and NGT, by diverse combinations of amino acids, the model comprising Arg turned out to be optimal. By analyzing the statistical model that we obtained, it could be concluded that Arg can be a potential biomarker of GDM and may be used for earlier detection of this disease. Since the variation in Arg concentration during pregnancy and postpartum is not statistically significant, it cannot unambiguously determine the participation of Arg in the normalization processes after pregnancy. We also tried to assess the influence of the pregnancy on metabolic transition profile in the GDM women by comparing postpartum results with data from the HMDB database, and we can state that only three amino acids, including Arg, Ile, and Phe, had comparable values with the data from HMDB. Taken together, the data of the presented work suggest that during pregnancy, alteration in amino acids profile may take place. Further investigations are needed in order to explain the role of Arg in the pathophysiology of GDM.

## Figures and Tables

**Table 1:** Anthropometric and biochemical characteristics of pregnant patients involved in the study assigned to two study groups: normoglycemic (NGT) and gestational diabetes mellitus (GDM). Data are presented as the median and interquartile range (25–75 percentiles).

Variable	NGT (*n* = 35)	GDM (*n* = 29)	*p*-Value
Age [years]	28.0 (26.0–31.0)	30.0 (28.0–34.0)	0.124
Pre-pregnancy BMI [kg/m^2^]	23.9 (21.8–26.7)	23.8 (20.2–26.3)	0.728
Gestational weight gain [kg]	8.0 (5.8–11.0)	9.2 (6.0–11.6)	0.401
FPG [mg/dL]	80.0 (75.0–85.0)	85.0 (81.0–101.0)	**0.002**
2h OGTT [mg/dL]	139.0 (139.0–125.0)	154.0 (145.0–166.0)	**0.000**
HbA1C [%]	5.1 (5.0–5.5)	5.1 (5.0–5.3)	0.799
insulin [µlU/mL]	8.0 (6.1–9.0)	12.1 (7.3–15.8)	**0.020**
HOMA-B	149.1 (122.4–174.5)	165.4 (139.8–241.9)	0.242
HOMA-IR	1.6 (1.1–1.8)	2.6 (1.5–3.7)	**0.005**
TG [mg/dL]	208.5 (174.5–246.8)	214.3 (172.7–227.0)	0.779
TC [mg/dL]	268.2 (230.5–288.3)	245.2 (224.2–275.6)	0.146
HDL-C [mg/dL]	83.3 (71.8–98.0)	75.2 (65.8–87.3)	0.120
LDL-C [mg/dL]	141.0 (121.0–168.0)	126.0 (116.0–150.0)	0.203
CRP [mg/L]	3.7 (2.1–7.3)	3.8 (2.1–5.3)	0.569

Abbreviations: BMI, body mass index; CRP, C reactive protein; FPG, fasting plasma glucose; HDL, high-density; lipoprotein; HbAC1-glycated haemoglobin; HOMA-B homeostatic model assessment to quantify beta-cell function; HOMA-IR, homeostasis model assessment of insulin resistance; LDL, low density lipoprotein; TC, total cholesterol; TGs, triglycerides. Values in bold are statistically significant.

**Table 2:** Comparison of plasma amino acids concentrations (µM/L) in normoglycemic (NGT) pregnant women and GDM pregnant women with logistic regression and ROC curve analysis. Concentration data for compared groups shown as medians and 25–75% quartiles. Fold changes based on medians. Univariate logistic regressions results shown as odd ratios with 95% confidence intervals, considering GDM as a modeled group.

AA ^$^	NGT (*n* = 35)	GDM (*n* = 29)	FC	*p*-Value (U Mann–Whitney Test)	OR (95%CI)	*p*-Value (LR)	*p*-Value (Wald’s Test)	ROC AUC
Ala	209.5 (195.0–243.0)	213.2 (192.3–232.5)	1.02	0.686	0.998 (0.987–1.009)	0.699	0.701	0.530
**Arg**	96.1 (82.3–116.6)	78.0 (62.6–90.4)	0.81	**0.001 #**	0.96 (0.934–0.986)	**0.000 #**	**0.003**	0.749
Asn	23.8 (21.8–26.8)	25.2 (21.7–27.5)	1.06	0.671	1.035 (0.94–1.139)	0.486	0.489	0.532
**Gln**	1054.1 (883.2–1127.3)	924.4 (807.8–1014.0)	0.88	**0.018**	0.997 (0.993–0.999)	**0.024**	**0.032**	0.673
Glu	74.4 (65.6–86.6)	76.2 (65.9–82.4)	1.02	0.656	0.988 (0.957–1.019)	0.443	0.448	0.533
Gly	113.5 (102.1–128.5)	110.0 (97.2–127.9)	0.97	0.458	0.995 (0.975–1.015)	0.587	0.590	0.555
**His**	132.2 (123.1–144.3)	119.9 (107.6–130.0)	0.91	**0.007**	0.968 (0.941–0.995)	**0.013**	**0.020**	0.698
Ile	43.2 (39.3–49.4)	41.5 (36.0–44.8)	0.96	0.142	0.955 (0.892–1.023)	0.172	0.187	0.608
Leu	107.1 (98.7–122.7)	107.0 (96.8–115.7)	0.99	0.345	0.987 (0.962–1.012)	0.295	0.309	0.569
Lys	136.3 (115.9–149.2)	126.0 (103.5–138.5)	0.92	0.084	0.986 (0.966–1.007)	0.169	0.183	0.627
**Met**	14.9 (13.3–17.8)	14.5 (12.3–15.7)	0.97	**0.010**	0.882 (0.747–1.042)	0.113	0.139	0.621
**Phe**	54.8 (49.5–61.9)	52.1 (46.4–55.2)	0.95	**0.019**	0.949 (0.895–1.006)	0.051	0.081	0.672
Pro	88.0 (77.6–101.7)	81.1 (75.6–92.2)	0.92	0.318	0.987 (0.963–1.012)	0.311	0.320	0.573
Pro-OH	6.7 (4.1–9.6)	8.0 (6.8–8.4)	1.19	0.366	1.081 (0.933–1.253)	0.281	0.297	0.566
**Ser**	99.6 (83.8–112.1)	85.9 (73.5–96.9)	0.86	**0.012**	0.963 (0.934–0.993)	**0.010**	**0.016**	0.684
Thr	108.1 (91.0–122.4)	109.8 (94.2–123.3)	1.02	0.696	1.005 (0.982–1.029)	0.651	0.652	0.529
Trp	60.3 (54.8–65.7)	59.7 (54.0–64.3)	0.99	0.866	0.999 (0.949–1.053)	0.983	0.983	0.513
Tyr	26.8 (24.3–30.9)	26.1 (22.5–27.6)	0.97	0.134	0.938 (0.855–1.029)	0.144	0.177	0.610
Val	121.7 (110.8–134.5)	129.1 (115.8–139.3)	1.06	0.418	1.008 (0.988–1.028)	0.449	0.455	0.560

^$^ Abbreviations: AA, amino acids; FC, fold change; LR, likelihood ratio; OR, odds ratio defined as the likelihood that an event will occur, expressed as a proportion of the likelihood that the event will not occur; ROC, receiver operating characteristic; AUC, area under the curve. # Significant differences after Benjamini–Hochberg correction. Values in bold are statistically significant.

**Table 3:** Spearman correlations of arginine plasma concentration (µM/L) with anthropometric and biochemical characteristics (including amino acids) in the entire studied group of patients during pregnancy (*n =* 64).

Variable	Rho	*p*-Value	FDR
Age [years]	−0.08	0.549	0.676
BMI [kg/m^2^]	0.14	0.275	0.400
Weight gein [kg]	−0.01	0.909	0.909
FPG [mg/dL]	−0.27	**0.032**	0.060
2h OGTT [mg/dL]	−0.27	**0.034**	0.060
HbA1C [mmol/mol]	0.03	0.813	0.839
Insulin [µlU/mL]	0.16	0.261	0.398
HOMA-B	0.31	**0.030**	0.060
HOMA-IR	0.09	0.515	0.659
TG [mg/dL]	0.33	**0.011**	**0.027**
TC [mg/dL]	0.05	0.692	0.764
HDL-C [mg/dL]	−0.14	0.308	0.429
LDL-C [mg/dL]	0.06	0.671	0.764
CRP [mg/L]	0.19	0.194	0.310
Ala [µM/L]	0.38	**0.002**	**0.007**
Asn [µM/L]	0.07	0.587	0.695
Gln [µM/L]	0.04	0.749	0.799
Glu [µM/L]	0.44	**0.000**	**0.001**
Gly [µM/L]	0.32	**0.011**	**0.027**
His [µM/L]	0.54	**0.000**	**0.000**
Ile [µM/L]	0.46	**0.000**	**0.001**
Leu [µM/L]	0.54	**0.000**	**0.000**
Lys [µM/L]	0.34	**0.006**	**0.018**
Met [µM/L]	0.61	**0.000**	**0.000**
Phe [µM/L]	0.81	**0.000**	**0.000**
Pro [µM/L]	0.38	**0.002**	**0.007**
Pro-OH [µM/L]	0.26	**0.041**	0.069
Ser [µM/L]	0.51	**0.000**	**0.000**
Thr [µM/L]	0.09	0.462	0.617
Trp [µM/L]	0.30	**0.017**	**0.040**
Tyr [µM/L]	0.66	**0.000**	**0.000**
Val [µM/L]	0.27	**0.033**	0.060

Abbreviations: see Table 2. Values in bold are statistically significant.

**Table 4:** Biochemical characteristics of the follow-up GDM group in two time points: 3 months after delivery (3 Mo) and 1 year postpartum (1 Y). Data compared to the 3rd trimester of pregnancy time point and expressed as a fold-change (FC).

Variable	3 Mo	FC *	1 Y	FC *	3Mo/GDM-1Y/3Mo Tendency	*p*-Value (Friedman’s Test)
FPG [mg/dL]	87.0 (82.0–91.0)	1.02	91.0 (82.0–101.0)	1.07	→-↗	**0.001 #**
2h OGTT [mg/dL]	96.0 (82.0–108.0)	**0.62**	101.5 (84.0–107.0)	**0.66**	↘-→	**0.000 #**
HbA1C [%]	5.2 (5.0–5.5)	1.02	5.1 (4.9–5.3)	1.00	→-→	0.094
insulin [µlU/mL	7.9 (5.9–10.0)	**0.65**	9.3 (5.5–11.1)	**0.77**	↘-→	**0.000 #**
HOMA-B	127.3 (94.2–178.1)	0.77	121.5 (89.1–168.0)	0.73	→-→	**0.003 #**
HOMA-IR	1.6 (1.1–2.3)	0.62	2.2 (1.1–2.7)	0.85	→-→	**0.004 #**
TG-C [mg/dL]	81.4 (56.9–102.3)	**0.38**	89.6 (53.9–103.1)	**0.42**	↘-→	**0.000 #**
TC [mg/dL]	187.9 (172.5–214.3)	**0.77**	185.0 (165.0–202.0)	**0.75**	↘-→	**0.000 #**
HDL-C [mg/dL]	61.1 (51.2–75.3)	**0.81**	63.0 (54.0–78.0)	**0.84**	↘-→	**0.001 #**
LDL-C [mg/dL]	115.0 (100.0–127.0)	**0.91**	95.0 (87.0–132.0)	**0.75**	↘-→	**0.018 #**
CRP [mg/L]	1.4 (0.7–2.6)	**0.37**	1.1 (0.8–1.9)	**0.29**	↘-→	**0.000 #**
Ala [µM/L]	266.4 (205.8–300.0)	**1.25**	237.3 (205.8–274.5)	1.11	↗-→	**0.006 #**
Arg [µM/L]	85.2 (76.5–99.1)	1.09	71.4 (63.1–81.1)	0.92	→-→	0.065
Asn [µM/L]	24.7 (23.7–26.6)	0.98	29.8 (26.3–32.5)	**1.18**	→-↗	**0.009 #**
Gln [µM/L]	1258.6 (1161.0–1450.5)	**1.36**	1323.8 (1179.1–1472.7)	**1.43**	↗-→	**0.000 #**
Glu [µM/L]	91.5 (78.9–99.0)	**1.20**	86.5 (72.2–96.0)	**1.14**	↗-→	**0.001 #**
Gly [µM/L]	229.0 (178.1–267.9)	**2.08**	196.3 (165.9–266.2)	**1.78**	↗-→	**0.000 #**
His [µM/L]	109.2 (96.5–123.7)	**0.91**	124.8 (113.1–136.7)	1.04	↘-↗	**0.032 #**
Ile [µM/L]	49.5 (40.2–58.9)	**1.19**	49.8 (46.0–54.6)	**1.20**	↗-→	**0.001 #**
Leu [µM/L]	138.2 (119.1–156.3)	**1.29**	137.2 (122.4–150.0)	**1.28**	↗-→	**0.000 #**
Lys [µM/L]	141.8 (128.9–151.3)	**1.13**	159.6 (132.1–185.1)	**1.27**	↗-→	**0.002 #**
Met [µM/L]	16.8 (14.7–17.8)	**1.16**	16.2 (14.5–18.7)	**1.12**	↗-→	**0.003 #**
Phe [µM/L]	58.1 (52.7–62.9)	**1.12**	58.3 (54.4–61.0)	**1.12**	↗-→	**0.000 #**
Pro [µM/L]	128.4 (116.3–145.4)	**1.58**	121.0 (101.9–145.8)	**1.49**	↗-→	**0.000 #**
Pro-OH [µM/L]	10.8 (7.0–15.5)	**1.35**	8.6 (6.7–10.6)	1.08	↗-→	**0.028 #**
Ser [µM/L]	92.2 (85.8–114.6)	**1.07**	116.6 (110.8–127.1)	**1.36**	↗-↗	**0.002 #**
Thr [µM/L]	64.6 (53.4–86.5)	**0.59**	84.3 (68.1–90.3)	**0.77**	↘-↗	**0.000 #**
Trp [µM/L]	66.5 (57.3–73.7)	**1.11**	75.8 (69.8–79.8)	**1.27**	↗-↗	**0.000 #**
Tyr [µM/L]	41.2 (36.3–47.8)	**1.58**	41.4 (37.4–44.3)	**1.59**	↗-→	**0.000 #**
Val [µM/L]	177.5 (152.8–196.8)	**1.37**	178.8 (159.9–196.6)	**1.38**	↗-→	**0.000 #**

Abbreviations: see Table 2. * Significant differences from pregnancy time point assessed by Wilcoxon test shown in bolded FC. * Comparisons among pregnancy three months postpartum and one year postpartum time points; *p*-values assessed by Friedman’s ANOVA. # Significant after Benjamini–Hochberg correction with alpha *=* 0.05; →, no change; ↗, increase;↘, decrease. Values in bold are statistically significant.

**Table 5:** Amino acids concentrations (µM/L) in plasma of studied patients in follow-up GDM group compared to NGT group.

AA	FC 3 Mo/NGT	*p*-Value	FC 1Y/NGT	*p*-Value
Ala	1.27	0.004 #	1.13	0.075
Arg	0.89	0.067	0.74	0.000 #
Asn	1.04	0.517	1.25	0.001 #
Gln	1.19	0.000 #	1.26	0.000 #
Glu	1.23	0.003 #	1.16	0.075
Gly	2.02	0.000 #	1.73	0.000 #
His	0.83	0.000 #	0.94	0.080
Ile	1.15	0.071	1.15	0.002 #
Leu	1.29	0.000 #	1.28	0.000 #
Lys	1.04	0.269	1.17	0.008 #
Met	1.13	0.169	1.09	0.094
Phe	1.06	0.526	1.06	0.215
Pro	1.46	0.000 #	1.38	0.000 #
Pro-OH	1.61	0.001 #	1.28	0.133
Ser	0.93	0.647	1.17	0.000 #
Thr	0.60	0.000 #	0.78	0.000 #
Trp	1.10	0.018 #	1.26	0.000 #
Tyr	1.54	0.000 #	1.54	0.000 #
Val	1.46	0.000 #	1.47	0.000 #

*p*-values assessed by U Mann–Whitney test; *#* Significant differences after Benjamini–Hochberg correction.

**Table 6:** Comparison of mean plasma concentrations of AA in NGT. One year postpartum group with external data (HMDB) [33] concerning the pregnant and non-pregnant healthy women.

AA	Preg. NGT	Preg. HMDB	FC	1 Y Post-GDM	Norm. HMDB	FC
ALA	217.5 (42.0)	258.6 (85.4)	0.84 *	244.4 (58.4)	319.4 (71.7)	0.77 *
ARG	99.7 (27.7)	122.8 (39.7)	0.81 *	73.9 (18.3)	75.0 (24.0)	0.98
ASN	24.3 (5.4)	31.9 (13.6)	0.76 *	29.8 (7.5)	47.0 (9.0)	0.63 *
GLN	1019.5 (180.4)	250.2 (77.3)	4.07 *	1323.8 (290.5)	578.0 (85.0)	2.29 *
GLU	79.3 (18.2)	NA		91.3 (26.7)	46.0 (13.0)	1.98 *
GLY	116.2 (22.3)	209.8 (74.9)	0.55 *	215.1 (65.9)	258.0 (64.0)	0.83 *
HIS	132.2 (19.2)	NA		122.4 (21.4)	83.0 (14.0)	1.47 *
ILE	44.4 (7.0)	40.9 (11.7)	1.09 *.	50.4 (7.0)	56.0 (12.0)	0.90
LEU	112.4 (19.9)	71.6 (40.6)	1.57 *	138.0 (22.1)	160.0 (27.0)	0.86 *
LYS	134.8 (24.4)	97.4 (29.5)	1.38 *	154.5 (37.7)	183.0 (34.0)	0.84 *
MET	15.8 (3.4)	26.1 (11.3)	0.61 *	16.8 (3.1)	27.0 (5.0)	0.62 *
PHE	57.1 (10.5)	58.2 (28.7)	0.98	58.5 (7.3)	52.0 (5.0)	1.12 *
PRO	92.2 (21.8)	125.4 (48.5)	0.73 *	127.0 (38.5)	168.0 (49.0)	0.76 *
H-Pro	7.2 (3.4)	NA		9.6 (6.8)	16.0 (9.0)	0.60 *
SER	97.4 (18.8)	132.0 (50.6)	0.74 *	115.4 (19.6)	127.0 (29.0)	0.91
THR	107.4 (20.1)	108.8 (42.5)	0.99	84.3 (22.2)	154.0 (40.0)	0.55 *
TRP	59.4 (9.8)			74.4 (11.2)	48.7 (11.6)	1.53 *
TYR	28.3 (6.7)	55.8 (19.6)	0.51 *	41.4 (7.6)	61.0 (13.0)	0.68 *
VAL	124.7 (22.8)	119.6 (38.1)	1.04	178.8 (35.4)	209.0 (31.0)	0.86 *

* *p* < 0.05 as assessed by *t* test.

**Table 7:** Inclusion criteria used for the patients’ participation in the study.

- diagnosis of GDM, - Caucasian ethnic background, - age range between 18 and 40 years, no family history of diabetes in first-degree relatives, - no GDM in a previous pregnancy, - absence of any form of pre-pregnancy diabetes.	- absence of concomitant systemic disease (chronic or acute or infectious), - no taking insulin or oral hypoglycemic medications, - no control by diet and exercise before the overnight fast.

## Supplementary Materials

Supplementary Materials can be found at https://www.mdpi.com/1422-0067/21/21/7811/s1. Figure S1: Data transformation and normalization prior to clustering and PCA analysis; Figure S2: Principal components (PCs) derived from the amino acids variables.

## Abbreviations

AAs	Amino Acids
FPG	Fasting Plasma Glucose
GDM	Gestational Diabetes Mellitus
HCA	Hierarchical Cluster Analysis
IGT	Impaired Glucose Tolerance
IR	Insulin Resistance
IUGR	Intrauterine Growth Restriction
NGT	Normal Glucose Tolerance
NO	Nitric Oxide
OGTT	Oral Glucose Tolerance Test
PCA	Principal Component Analysis
T2DM	Type 2 Diabetes Mellitus
