# Supplementary material for: Associations of Arginine with Gestational Diabetes Mellitus in a Follow-Up Study
Source: Int J Mol Sci. 2020 Oct 22;21(21):7811. doi: 10.3390/ijms21217811 (PMC7659483; doi:10.3390/ijms21217811)
Supplement: Supplementary file 1 [file ijms-21-07811-s001.pdf]

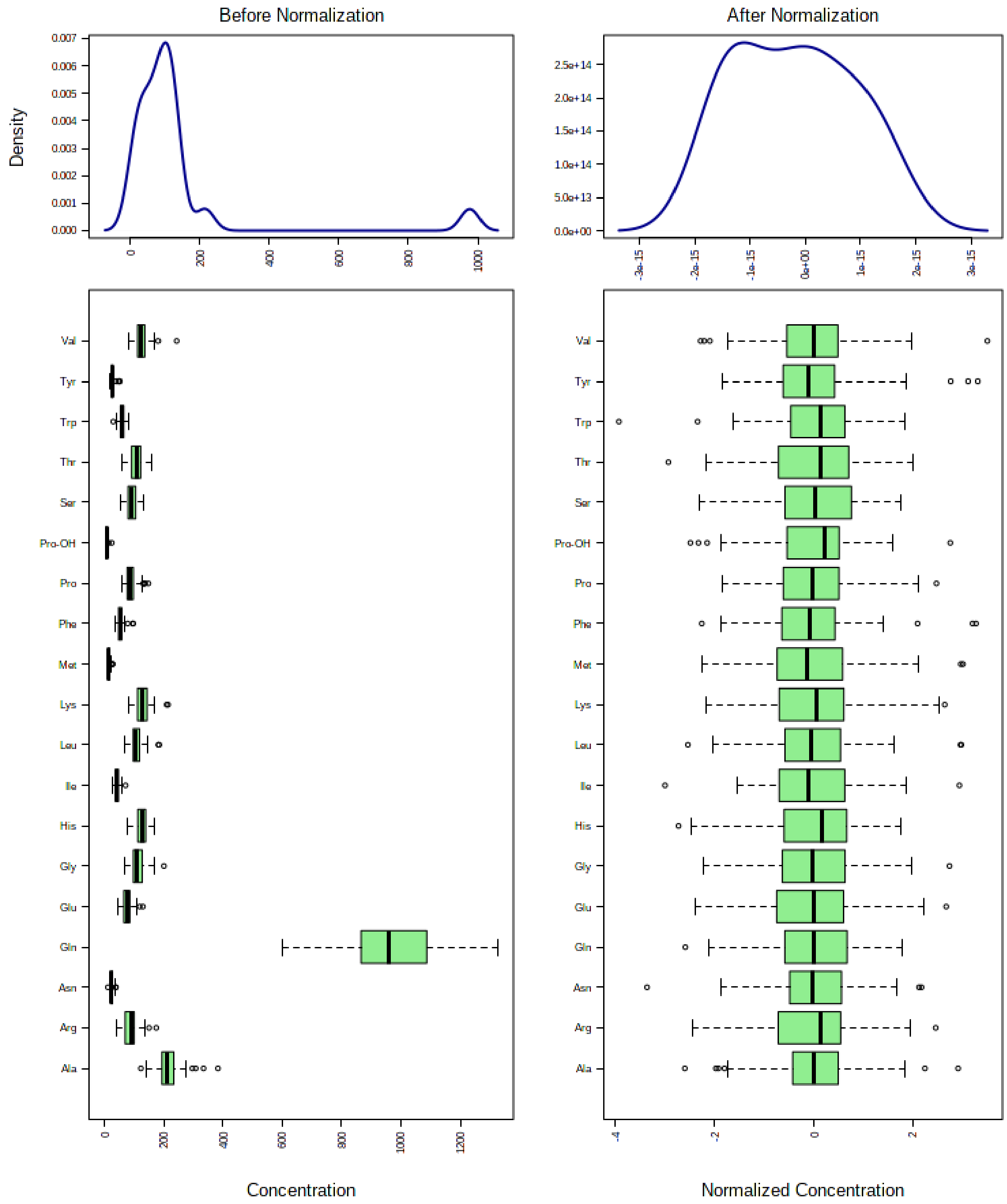

Supplementary Figure S1. Data transformation and normalization prior to clustering and PCA analysis. Box plots and kernel density plots before and after normalization. The density plots are based on all samples. Selected methods: Row-wise normalization: N/A; Data transformation: Log Normalization; Data scaling: Autoscaling.

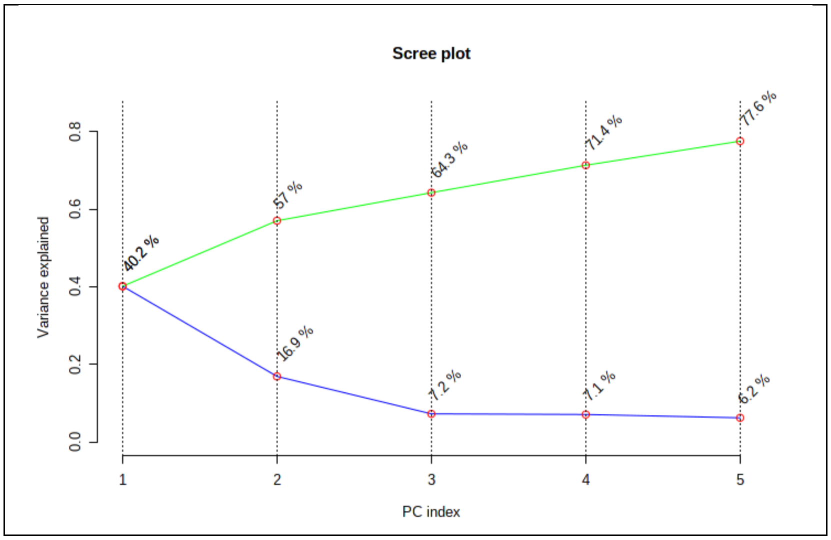

Supplementary Figure S2. Principal components (PCs) derived from the amino acids variables. Scree plot showing the variance explained by PCs. The green line on top shows the accumulated variance explained; the blue line underneath shows the variance explained by individual PC.
